# Is Ultrasonography an Effective Method for Diagnosing Degenerative Changes in the Temporomandibular Joint?

**DOI:** 10.3390/biomedicines12122915

**Published:** 2024-12-21

**Authors:** Barbara Wojciechowska, Arkadiusz Szarmach, Adam Michcik, Maciej Sikora, Barbara Drogoszewska

**Affiliations:** 1 Department of Maxillofacial Surgery, Medical University of Gdansk, 17 Mariana Smoluchowskiego Street, 80-214 Gdansk, Poland; adammichcik@gumed.edu.pl (A.M.); drog@gumed.edu.pl (B.D.); 2 2nd Department of Radiology, Medical University of Gdansk, 17 Mariana Smoluchowskiego Street, 80-214 Gdansk, Poland; 3 National Medical Institute of the Ministry of Interior and Administration, 137 Wołoska Street, 02-507 Warsaw, Poland; sikora-maciej@wp.pl; 4 Department of Maxillofacial Surgery, Hospital of the Ministry of Interior, 51 Wojska Polskiego Street, 25-375 Kielce, Poland; 5 Department of Biochemistry and Medical Chemistry, Pomeranian Medical University, 72 Powstanców Wielkopolskich Street, 70-111 Szczecin, Poland

**Keywords:** temporomandibular joint disorders, ultrasonography, cone-beam computed tomography, osteoarthritis

## Abstract

Background: The accurate diagnosis of degenerative joint diseases (DJDs) of the temporomandibular joint (TMJ) presents a significant clinical challenge due to their progressive nature and the complexity of associated structural changes. These conditions, characterized by cartilage degradation, subchondral bone remodeling, and eventual joint dysfunction, necessitate reliable and efficient imaging techniques for early detection and effective management. Cone-beam computed tomography (CBCT) is widely regarded as the gold standard for evaluating osseous changes in the TMJ, offering detailed visualization of bony structures. However, ultrasonography (US) has emerged as a promising alternative, offering a non-invasive and radiation-free option for assessing TMJ disorders. This study aims to evaluate the diagnostic accuracy of US in identifying degenerative changes in the TMJ, with CBCT serving as the definitive diagnostic reference. By analyzing the sensitivity, specificity, and predictive values of US in detecting key degenerative markers—such as subchondral erosion, osteophytes, and joint space narrowing—this investigation seeks to assess its utility as a screening tool and its potential integration into clinical workflows. Methods: Forty adult patients presenting temporomandibular joint disorders were included in our cross-sectional study. Each patient underwent a clinical examination and was subjected to cone-beam computed tomography (CBCT) and ultrasonography (US). A statistical analysis was performed to compare the imaging results from CBCT and US. Results: The results are summarized in three tables. The first table presents a comparative analysis of radiological outcomes in patients with temporomandibular joint disorders using different imaging techniques. CBCT demonstrated higher sensitivity in detecting osteophytes in the right mandibular head (27.50% vs. 7.50%, *p* = 0.027) and higher detection rates for erosions, though without a significant advantage over US. The second table analyzes the consistency of diagnostic results between CBCT and US. A moderate agreement was observed for detecting normal bone structures, with AC1 values of 0.58 for the right and 0.68 for the left mandibular head (*p* < 0.001). The third table evaluates the diagnostic accuracy of US compared to CBCT. US demonstrated a positive predictive value (PPV) of 90% for detecting normal conditions, indicating its high reliability as a screening tool for normal findings. US demonstrates higher effectiveness in ruling out certain issues due to its high specificity and negative predictive value. However, its lower sensitivity in detecting abnormalities may lead to both false-positive and false-negative results. Conclusions: US holds significant promise as a screening modality for detecting normal anatomical features of the temporomandibular joint, its limitations in identifying more complex degenerative changes necessitate a cautious and integrated approach to TMJ diagnostics.

## 1. Introduction

The temporomandibular joint (TMJ) is a paired joint that operates synergistically as part of the stomatognathic system [1,2]. It is composed of the head of the condylar process, the articular disc, the articular fossa located on the temporal bone, and a reinforcing capsule with ligaments [3,4]. The articular disc is made of fibrous connective tissue and divides the temporomandibular joint into two compartments: upper and lower. It takes on a biconcave or oval shape [1,4]. The average thickness of the articular disc is 2 mm in the anterior part, 3 mm in the posterior part, and 1 mm in the central part [1,2,4]. The condylar process of the mandible has an elliptical shape. It measures 15 to 20 mm from side to side and 8 to 10 mm from front to back. There are several classifications of the shape of the mandibular condylar head, with the one most cited in the literature: rounded, angled, flattened, and mixed [2]. Within the temporomandibular joint, hinge and sliding movements occur, facilitated by the movement of the articular disc. Hinge movements take place in the lower compartment, while sliding movements occur in the upper compartment [2]. The morphology of the condylar process changes throughout life due to developmental alterations, remodeling, diseases, injuries, endocrine disorders, and radiotherapy [5]. The functions of the temporomandibular joints include mastication, chewing, swallowing, phonation, breathing, and facial expressions [1,6].

Temporomandibular disorders (TMDs) encompass a wide spectrum of musculoskeletal issues that impact the temporomandibular joint, as well as the surrounding masticatory muscles and other related anatomical structures [7,8]. These disorders can cause a variety of symptoms, including jaw pain, difficulty chewing, and clicking or popping sounds in the TMJ. Up to 34% of the population may experience some form of TMD, with a notably higher prevalence among women compared to men [9]. The conditions are most observed between the ages of 30 and 50, although they can affect individuals outside this age range as well. Research also shows that the severity and frequency of TMD symptoms tend to increase with age, suggesting a progressive component to these disorders [8,9,10,11].

One of the conditions of TMD is Degenerative Joint Disease (DJD), a progressive degenerative condition of the temporomandibular joint, characterized by cartilage degradation and bone remodeling [12]. DJD includes osteoarthritis and osteoarthrosis. These two conditions differ in the accompanying pain symptoms—TMJ osteoarthritis (TMJ OA) indicates degenerative changes with pain in the joint whereas TMJ osteoarthrosis is a degenerative condition without any pain-related symptoms [13]. TMJ OA is characterized by a gradual and progressive destruction of the articular tissues, including the articular cartilage, subchondral bone, synovial membrane, and adjacent soft tissues. This process leads to the thinning and eventual loss of articular cartilage [14,15,16]. TMJ degenerative joint disease typically progresses through three phases, with periods of remission and repair in between. The initial phase is marked by TMJ clicking and occasional locking, while the intermediate phase is characterized by TMJ pain, difficulty, or limitation in opening the mouth, and crepitus. In the final phase, degenerative activity subsides, leading to relative joint stability [17]. As the cartilage deteriorates, the underlying subchondral bone becomes exposed and may undergo sclerosis, cyst formation, and the development of osteophytes or bone spurs. These changes contribute to altered joint mechanics, pain, and decreased range of motion [12]. The synovial membrane, responsible for producing synovial fluid that lubricates the joint, may also become inflamed and thickened, exacerbating the symptoms and progression of the disease. The cumulative effect of these pathological changes results in joint dysfunction, significant pain, and impaired quality of life for affected individuals. With advanced degeneration, the subchondral cortical layer is lost, and erosion and other radiographic signs of osteoarthritis (OA) appear [18].

TMJ OA affects the condylar head, articular eminence, and glenoid fossa. The most frequently observed changes in TMJ OA patients are condylar flattening and erosion of the articular surface. According to the DC/TMD guidelines, a diagnosis of DJD can be confirmed if cone-beam computed tomography (CBCT) imaging reveals at least one of the following: subchondral cysts, erosions, generalized sclerosis, or osteophytes [13,19]. The preferred examination used to assess the bone structures of the joint, and thus evaluate degenerative changes, is cone-beam computed tomography. CBCT provides high-resolution, three-dimensional images that allow for detailed visualization of the bony architecture. This is crucial in identifying subtle degenerative changes that might not be apparent on two-dimensional radiographs.

**Figure 1:**
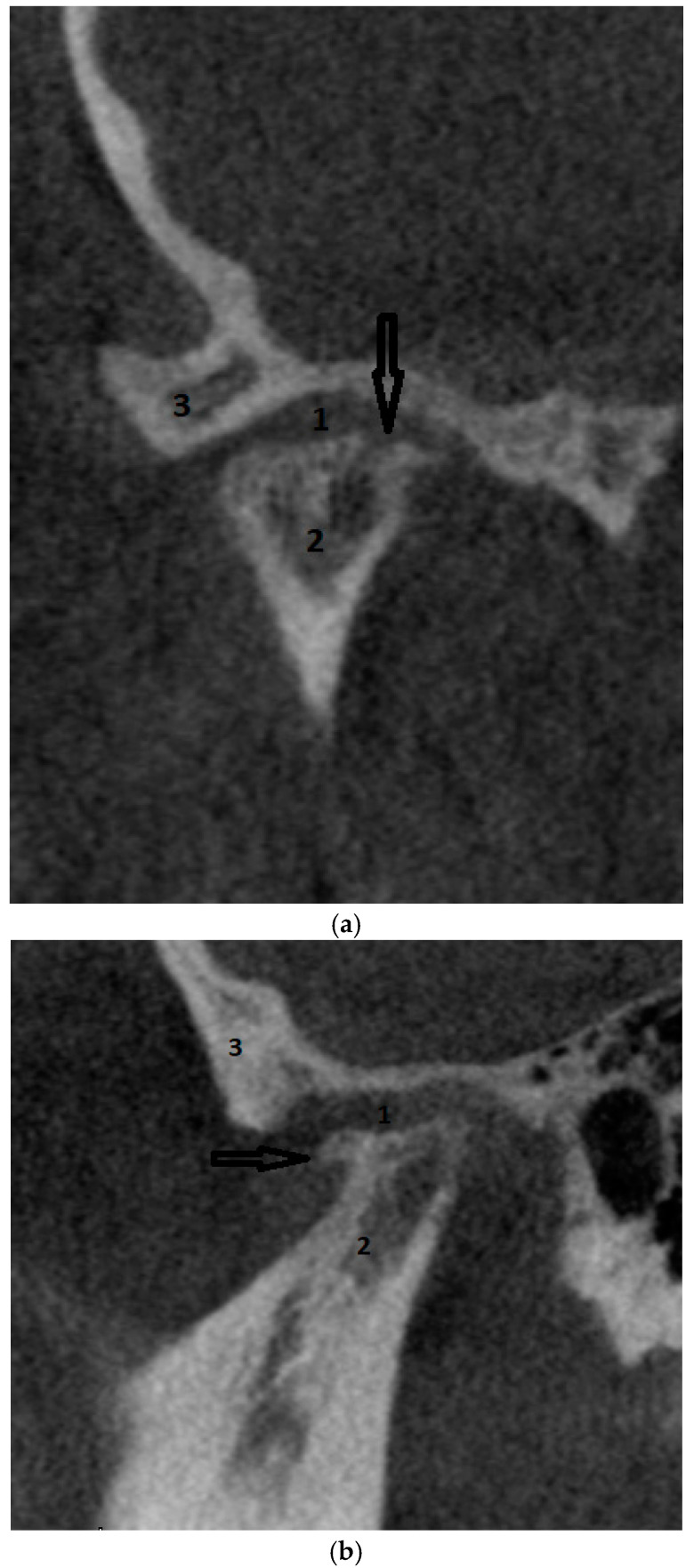
(**a**) CBCT (coronal view). Black arrow points to erosion and subchondral cyst. Anatomical structures of the TMJ: 1. Disc, 2. Condyle, 3. Temporal Bone; (**b**) CBCT (sagittal view). Black arrow indicates osteophyte. Anatomical structures of the TMJ: 1. Disc, 2. Condyle, 3. Temporal Bone.

The ability of CBCT to produce images with high spatial resolution and contrast helps in the detailed assessment of joint spaces, cortical bone integrity, and subchondral bone changes (Figure 1a,b). This makes CBCT particularly useful in diagnosing early stages of osteoarthritis and other degenerative joint diseases. Additionally, the multiplanar reconstruction capability of CBCT allows clinicians to view the joint in various planes without distortion, providing a comprehensive assessment that is not possible with conventional radiography. CBCT involves a relatively lower radiation dose compared to conventional computed tomography (CT) while still providing superior image quality. This is particularly important in clinical settings where repeated imaging might be necessary to monitor the progression of degenerative changes or the effectiveness of treatment interventions [10,20,21,22].

Magnetic resonance imaging (MRI) is a radiological technique widely used in the diagnosis of temporomandibular joint disorders and is considered the gold standard for evaluating disc-related pathologies. In contrast, computed tomography is regarded as the most effective method for visualizing bone structures and osteoarthritis, while MRI remains superior for assessing soft tissues, including the disc and its relationship within the joint [8,11].

There are few reports in the literature about the use of ultrasound (US) in the diagnosis of temporomandibular joints [1,8,15,18,19,20]. Ultrasound is a commonly used, affordable, non-invasive, and safe imaging technique that allows for real-time visualization [23]. The principal aim of this investigation was to evaluate the potential utility of ultrasonography as a diagnostic screening modality for identifying degenerative changes in the temporomandibular joints of adult patients, utilizing cone-beam computed tomography as the definitive diagnostic. We selected CBCT as the reference examination due to its high-resolution imaging capabilities which allow for detailed visualization of bone structures. This choice ensures accurate assessment and comparison of diagnostic outcomes, providing a robust standard against which to evaluate the effectiveness of ultrasound in detecting degenerative joint disease.

The TMJ is a complex structure essential for functions like chewing, swallowing, and speaking, with its morphology adapting to various physiological and pathological factors over time. Temporomandibular disorders are musculoskeletal conditions affecting the TMJ and surrounding structures, with higher prevalence among women and symptoms often worsening with age. Among these, DJD—including osteoarthritis (painful) and osteoarthrosis (non-painful)—is a progressive condition marked by cartilage degradation, bone remodeling, and synovial inflammation, leading to joint dysfunction and impaired quality of life. CBCT remains the preferred diagnostic method for identifying DJD, including early degenerative changes like subchondral cysts and osteophytes. This study evaluates the utility of US as a non-invasive, cost-effective screening tool for detecting TMJ degenerative changes, using CBCT as the definitive diagnostic reference.

## 2. Materials and Methods

Forty patients with temporomandibular joint disorders were enrolled in this cross-sectional study. All patients underwent a clinical examination, cone-beam computed tomography, and ultrasonography.

This study was conducted at the Department of Maxillofacial Surgery in the Medical University of Gdańsk. All radiological examinations were performed at the Department of Radiology at the Medical University of Gdańsk. Subjects gave consent to participate in the study. The study was approved by the Ethics Committee of the Medical University of Gdańsk.

The inclusion and exclusion criteria are presented in Table 1 [24].

In our radiological examination, we evaluated the anatomy of the mandibular head, focusing on the presence of erosions and osteophytes. We also assessed bone structure and derived conclusions based on the findings. All CBCT examinations were performed using a Carestream CS 9300 (Kodak Dental Systems, San Francisco, CA, USA). Each US examination was conducted by the same experienced radiologist using a LOGIQ E10 ultrasound machine equipped with a specialized L8-18i hockey stick linear probe (GE Healthcare, Boston, MA, USA). For this study, a unique ultrasound research protocol was developed to evaluate the temporomandibular joint. The protocol included assessments in three key conditions: the neutral position (with the mouth closed), maximum mouth opening, and the dynamic analysis of the articular disc’s movement path. Additionally, the protocol encompassed the evaluation of the bone structure outlines. All ultrasound images were obtained in two orthogonal projections—transverse and longitudinal—positioned at an angle of 90 degrees to each other.

### Statistical Analysis

Initially, a McNemar’s test was implemented to evaluate the presence of significant discrepancies in the symmetry of diagnostic outcomes between the CBCT and US modalities for the same cohort of TMJ patients. Subsequently, the consistency of radiological findings between US and CBCT was quantified using Gwet’s AC1 coefficient, providing a robust measure of inter-modality agreement that accounts for agreement occurring by chance. The final analytic phase involved comparing the diagnostic outcomes from US (as the predicted variable) with those from CBCT (as the observed variable).

The threshold for statistical significance was set at an alpha level of 0.05. To evaluate the distribution of numerical variables, the Shapiro–Wilk test was employed to determine normality. Variables found to deviate from a normal distribution were summarized using the median (Mdn) and the interquartile range, particularly noting the first (Q1) and third (Q3) quartiles. For categorical variables, summaries were provided in the form of counts (*n*) and percentages (%) for each category.

Analyses were conducted using the R Statistical language (version 4.3.1; R Core Team, 2023) on Windows 10 pro 64-bit (build 19045).

## 3. Results

The demographic breakdown revealed a predominant female representation, with 31 female participants (77.5%) and 9 male participants (22.5%). The median age of the participants was 38.5 years, with an interquartile range (IQR) from 23.75 to 56.50 years. Regarding the duration of symptoms prior to diagnosis, most of the participants, 25 out of 40 (62.5%), reported experiencing ailments related to the TMJ for more than six months. The remaining 15 participants (37.5%) had symptoms persisting for six months or less.

Within the examined cohort, CBCT scans and ultrasound evaluations underwent comprehensive analysis. The focus was on 12 distinct diagnostic parameters present in both imaging modalities, facilitating a direct comparison of their respective outcomes as detailed in Table 2.

US tends to yield higher detection rates for normal anatomical outlines in the right mandibular head compared to CBCT (77.50% vs. 55.00%, *p* = 0.039). CBCT shows a higher detection rate of abnormal outlines in the same region.

In the detection of osteophytes, CBCT exhibits a higher sensitivity in the right mandibular head (27.50% vs. 7.50%, *p* = 0.027). For erosions, neither technique shows a significant advantage, although CBCT still has higher detection rates.

When assessing the overall integrity of the bone structure, CBCT consistently shows higher rates of normalcy detection in both the right (90.00% vs. 75.00%, *p* = 0.149) and left mandibular heads (87.50% vs. 75.00%, *p* = 0.182).

We explored the consistency of diagnostic results obtained through two different radiological techniques: CBCT and US (Table 3). The level of agreement between these modalities across different radiological outcomes was assessed, including the delineation of normal and abnormal mandibular head outlines, the presence of erosions, osteophytes, and overall bone structure integrity.

Observation of the mandibular head’s normal and abnormal outlines, both the right and left sides exhibit low agreement levels, as indicated by AC1 values of 0.32 for normal and abnormal outlines on the right and even lower on the left with 0.06 for normal and 0.28 for abnormal outlines.

For erosions, the AC1 values are very high, 0.74 on the right and 0.79 on the left, both with *p* < 0.001. This trend is similarly observed with osteophytes where the agreement is substantial (0.65 on the right and 0.69 on the left), again with very low *p*-values (*p* < 0.001).

The evaluation of bone structure also showed a higher level of agreement, particularly normal bone structures with AC1 values of 0.58 on the right and 0.68 on the left (*p* < 0.001).

In the overall assessment of degenerative changes in both joints, the agreement levels drop to moderate (0.35 on the right and 0.41 on the left).

Table 4 systematically displays a comprehensive array of diagnostic accuracy that compares US outcomes as the test modality against the findings from CBCT as the observational standard. For the right mandibular head, the sensitivity of US in detecting a normal outline is quite high at 86%. The specificity is relatively low at 33%. This is counterbalanced by a positive predictive value of 61%, suggesting that when US indicates a normal outline, there is a reasonable probability that this is correct. The negative predictive value (NPV) of 67% further supports US’s role in effectively ruling out non-normal conditions when a normal result is obtained.

Contrastingly, US shows a lower sensitivity (33%) but higher specificity (86%) in detecting abnormal outlines.

**Figure 2:**
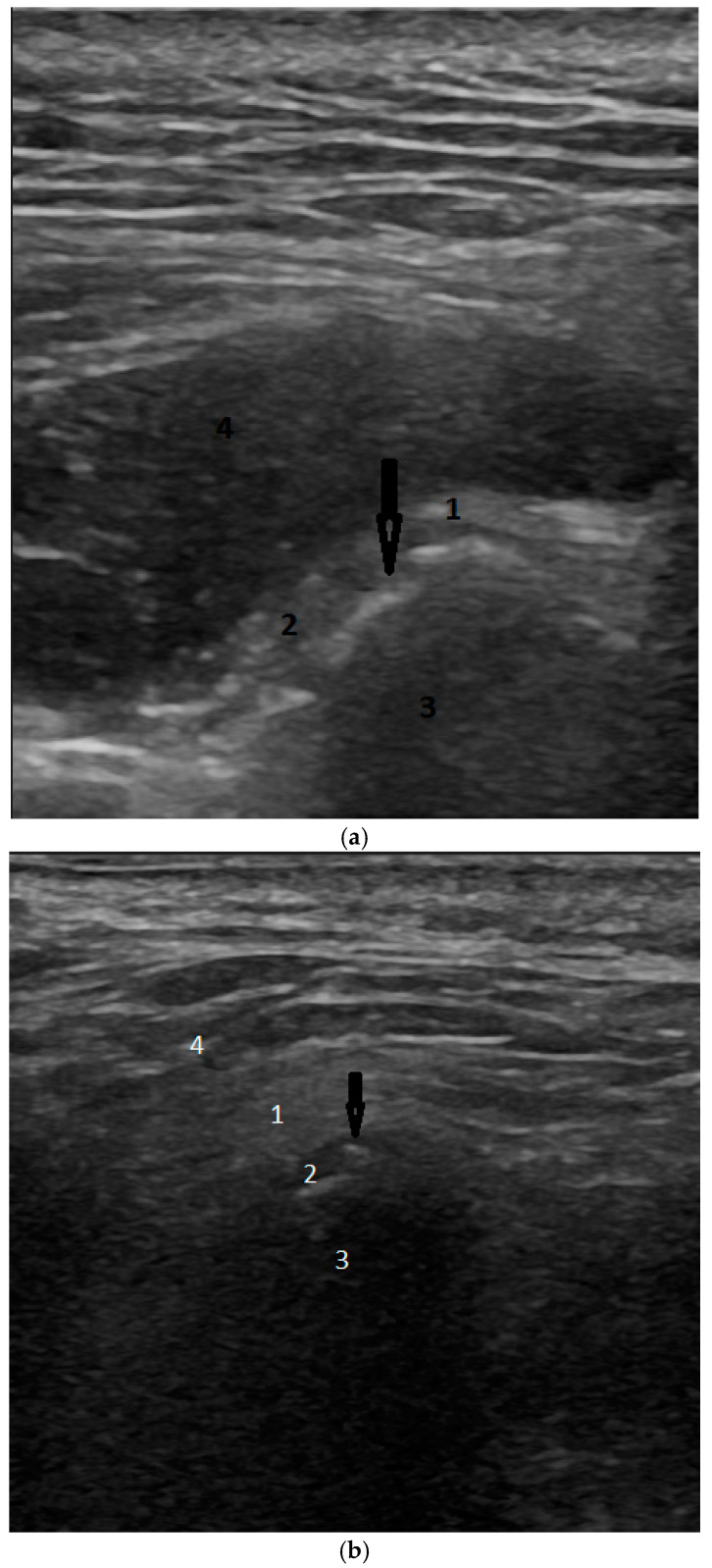
(**a**) Ultrasonography (sagittal view). Black arrow is pointing to erosion. Anatomical structures: 1. Glenoid Fossa, 2. Disc, 3. Condyle, 4. Masseter muscle; (**b**) Ultrasonography (axial view). Black arrow shows osteophyte. Anatomical structures: 1. Glenoid Fossa, 2. Disc, 3. Condyle, 4. Masseter muscle.

For erosions, ultrasound has very low sensitivity (14%) but very high specificity (94%). Regarding osteophytes, the sensitivity remains low (18%) while maintaining high specificity (97%), reflecting a similar pattern to that seen with erosions (Figure 2a,b).

In the left mandibular head, sensitivity and specificity for normal outlines are more balanced at 56% and 38%, respectively. Diagnostic metrics for abnormal outlines, erosions, and osteophytes on the left side follow similar trends to those on the right side, although with slight variations in sensitivity and specificity.

For normal bone structure on the right side, US has a sensitivity of 75%, but the specificity is notably low at 25%. For degenerative changes in the right joint, US demonstrates moderate sensitivity (63%) and better specificity (71%).

In the left joint, US shows a high sensitivity of 86%, indicating excellent ability to detect degenerative changes when present, though the specificity of 62% still poses a risk of false positives. The PPV is 55%, suggesting caution in interpreting positive results. However, the high NPV of 89% provides reliability in negative findings.

For detecting normal conditions, the PPV is 90%, meaning that a normal US result is highly reliable. Conversely, the NPV of 10% is very low, indicating that a US diagnosis of abnormality is often unreliable.

Table 4 serves as a pivotal resource for clinicians and researchers, offering a detailed analysis of the diagnostic effectiveness of ultrasonography in comparison to cone-beam computed tomography, which is regarded as a more definitive imaging modality. The metrics presented are essential for assessing the reliability of US in detecting and diagnosing mandibular abnormalities, thereby guiding clinical decisions and enhancing patient care strategies. Each metric is accompanied by its respective confidence interval, highlighting the statistical robustness and precision of the findings.

Table 4 not only encapsulates the comparative diagnostic accuracy of these two significant imaging techniques but also emphasizes their practical implications in clinical practice. This supports informed diagnostic and therapeutic interventions, reinforcing the importance of choosing the appropriate imaging modality based on specific clinical needs.

## 4. Discussion

Our study holds particular significance as it addresses a notable gap in the existing literature by providing insights into the diagnostic accuracy of ultrasonography compared to cone-beam computed tomography in detecting degenerative changes within the temporomandibular joint. The most recent meta-analysis by Zaman et al. [25] examined the effectiveness of US in diagnosing TMJ disorders but exclusively compared US with magnetic resonance imaging, leaving unexamined the potential of US in comparison to CBCT for diagnosing degenerative joint disease. By exploring this gap, our study offers a unique perspective on optimizing TMJ disorder diagnostics, which may directly impact clinical decision making and patient outcomes.

Consistent with the findings reported in the existing literature and observed in our study, most patients diagnosed with temporomandibular joint disorders were female. This gender predominance may be attributed to various factors, including hormonal influences, particularly the effects of estrogen, which has been implicated in the modulation of pain sensitivity and joint health. Additionally, the broad age distribution of the affected individuals suggests that TMJ disorders are not confined to a specific age group but rather manifest across a wide spectrum of ages. This observation is in line with other studies, which have demonstrated that TMJ disorders can develop at any stage of life, further emphasizing the multifactorial nature of these conditions and the potential impact of both biological and environmental factors throughout different life phases [9,10,17].

Imaging the TMJ using ultrasonography presents specific challenges for clinicians, including a limited field of examination, restricted accessibility to deeper structures, and the high likelihood of ultrasound reflections from bone tissue, all of which complicate image interpretation. The imaging protocol involves transverse and longitudinal scans of the anterosuperior joint compartment across coronal, axial, and oblique planes. A linear transducer (7.5–20 MHz) is positioned perpendicular to the zygomatic arch and parallel to the mandibular ramus, then adjusted to optimize visualization. Static and dynamic assessments are performed at different levels of mouth opening [26].

Cortical bone structures, including the condylar head and glenoid fossa, appear hyperechoic (white) on US, while bone marrow is hypoechoic (black), and connective and muscular tissues are isoechoic (gray). Fluid-filled spaces, including the superior and inferior joint compartments, are hypoechoic [23,26]. Despite these detailed visualizations, US presents challenges in accurately identifying more complex TMJ degenerative changes, as confirmed by our findings.

The diagnostic efficacy of cone-beam computed tomography has been shown to be comparable to that of conventional computed tomography. However, given the markedly lower radiation dose associated with CBCT, we elected to employ CBCT as the reference standard in our study [20].

We evaluated the level of agreement between CBCT and US across various radiological outcomes. This included the delineation of normal and abnormal mandibular head outlines, the presence of erosions and osteophytes, and the overall integrity of the bone structure.

The comparison between ultrasonography and cone-beam computed tomography in the diagnosis of temporomandibular joint disorders reveals notable differences in detection capabilities, particularly in identifying structural abnormalities such as osteophytes and erosions, which are key indicators of degenerative joint disease. CBCT demonstrates a higher detection rate of abnormal outlines in the TMJ region compared to US, which suggests superior specificity and a greater ability to confirm abnormalities when they are present. CBCT shows significantly higher sensitivity in detecting osteophytes on the mandibular head (*p* = 0.027), underscoring its potential advantage in cases where detailed imaging of bone changes is required. This enhanced sensitivity is due to CBCT’s higher spatial resolution and superior bone tissue differentiation capabilities [20]. In terms of erosion detection, while CBCT also exhibits higher detection rates, the advantage is not as pronounced as with osteophytes.

US demonstrates a high sensitivity in identifying a normal outline for the right mandibular head. This high sensitivity indicates that US is proficient in correctly identifying patients who do not have abnormalities, thus serving as an effective initial screening tool. However, the specificity of US in this context is relatively low at 33%. This low specificity suggests a significant rate of false positives, where US may inaccurately suggest a normal outline when abnormalities are present. Despite this limitation, the positive predictive value of US for detecting normal outlines is 61%. This means that when US indicates a normal outline, there is a moderate likelihood that this finding is accurate. The negative predictive value of 67% further emphasizes the utility of US in ruling out abnormalities when a normal result is obtained, indicating that a negative US result is a good predictor of the absence of disease. In contrast, US shows different performance metrics in detecting abnormal outlines. It has a lower sensitivity of 33%, which implies that US may miss a considerable number of true abnormalities potentially leading to underdiagnosis. However, the specificity increases to 86%, indicating that when US identifies an abnormal outline, it is likely correct. This high specificity is particularly valuable in clinical scenarios where the confirmation of abnormal findings is critical. In such cases, a positive US finding warrants further investigation with a more definitive imaging modality, such as cone-beam computed tomography, to confirm the diagnosis and inform treatment planning. These findings underscore the importance of understanding the strengths and limitations of US as a diagnostic tool. While it is effective for initial screening and excluding non-abnormal conditions, its variable sensitivity and specificity necessitate cautious interpretation of results. Consequently, integrating US findings with high-resolution imaging techniques like CBCT is advisable, especially in cases requiring a definitive diagnosis [27].

Our findings highlight the strengths and limitations of US as a diagnostic tool. While US is effective for initial screening and identifying normal anatomical features of the TMJ, its variable sensitivity and specificity warrant careful interpretation. A cautious and integrated approach is necessary, especially when dealing with complex TMJ pathologies. Specifically, US alone may not provide a comprehensive evaluation of intricate degenerative changes, and integrating US with CBCT as a definitive diagnostic standard is essential for enhancing diagnostic accuracy and reliability.

In clinical practice, these results support the adoption of a multimodal diagnostic strategy where US serves as an initial, non-invasive screening tool, complemented by CBCT in cases with ambiguous findings or complex conditions. Although US proves particularly useful for identifying normal bone structures and detecting certain degenerative changes, it should be applied judiciously with confirmatory CBCT imaging to ensure diagnostic precision and reliability.

Disorders of the temporomandibular joint can contribute to significant dysfunction and substantial limitations in quality of life [28,29]. Therefore, the use of readily accessible and reliable diagnostic methods is essential. The selection of an appropriate imaging modality, such as cone-beam computed tomography or ultrasound, for the evaluation of temporomandibular joint disorders, should be guided by specific clinical indications and the characteristics of the suspected pathology. US is a valuable diagnostic tool due to its high sensitivity for detecting normal anatomical structures, non-invasive nature, and absence of ionizing radiation, making it particularly advantageous for initial evaluations and routine screenings. However, its diagnostic performance is limited and highly operator-dependent, requiring extensive training and experience for accurate image acquisition and interpretation. This variability poses challenges in achieving consistent diagnostic accuracy and repeatability, highlighting the need for standardized training protocols [1,8,30].

CBCT, on the other hand, requires less operator-specific expertise due to its automated imaging process, resulting in higher repeatability and reliability. CBCT excels in visualizing bony structures, providing detailed insights into osseous changes such as erosions, osteophyte formation, and joint space alterations—key diagnostic markers of degenerative joint conditions. Its high spatial resolution and three-dimensional imaging capabilities make it indispensable for assessing bony pathologies. The associated ionizing radiation and limited capacity for soft tissue evaluation pose significant constraints, especially in younger patients or scenarios requiring repeated imaging [17,25,31,32,33]. Comparing the two modalities, while US offers significant advantages in terms of safety and accessibility, CBCT provides more consistent and repeatable results for diagnosing DJD, making it a more reliable choice for certain clinical situations.

When ultrasound findings are inconclusive or suggest the presence of more complex pathologies, follow-up imaging with cone-beam computed tomography is often necessary to ensure diagnostic accuracy. Future research should focus on optimizing diagnostic workflows by integrating the complementary strengths of US and CBCT to enhance the evaluation of temporomandibular joint disorders. Nonetheless, US alone cannot be considered a sufficient radiological examination for the precise evaluation of hard TMJ structures in pathological cases.

## 5. Conclusions

This study explored the potential of ultrasonography as an efficient screening tool for detecting degenerative changes in the temporomandibular joint, with cone-beam computed tomography serving as the diagnostic reference. The findings partially support this hypothesis, demonstrating that US has notable sensitivity (86%) and a high negative predictive value (89%) for identifying normal anatomical outlines, making it a valuable tool for ruling out TMJ abnormalities. However, US shows limited sensitivity for detecting key degenerative markers, such as osteophytes (18%) and erosions (14%), despite achieving high specificity (97% and 94%, respectively), highlighting the indispensable role of CBCT in achieving definitive diagnostic accuracy.

While US holds promise as a non-invasive, radiation-free modality for initial screening, its limitations in detecting subtle or complex degenerative changes necessitate cautious interpretation. A multimodal diagnostic strategy that integrates US with high-resolution imaging techniques, such as CBCT, ensures a more precise and comprehensive evaluation of TMJ pathologies. This approach not only optimizes diagnostic workflows but also enhances clinical decision-making and patient outcomes.

The choice between CBCT and US should depend on the specific clinical context. US is particularly advantageous for its accessibility and ability to identify normal anatomical structures with high sensitivity. However, in cases of inconclusive findings or suspected complex pathology, CBCT is recommended to provide the detailed imaging necessary for accurate diagnosis. This complementary use of both modalities offers a balanced, patient-centered approach to managing TMJ disorders.

## Figures and Tables

**Table 1:** Inclusion and exclusion criteria.

Inclusion Criteria	Exclusion Criteria
Patients over 18 years of age	Patients under 18 years of age
Patients who presented to the Maxillofacial Surgery Department due to TMD	Patients with polyarthritis (such as rheumatoid arthritis, gout arthritis and psoriatic arthritis)
Patients who agreed to participate in the study	Subjects who were not willing to participate in the study
	Patients with history of TMD treatment
	Patients with trauma
	Patients with a history of orthodontic treatment, plastic surgery, or other craniofacial surgery

**Table 2:** Comparative analysis of the radiological outcomes in patients diagnosed with temporomandibular joint disorder by the examination technique.

Characteristic	*n*	Examination Technique		*p ^b^*
		*CBCT*, *n*_1_ = 40 ^*a*^	*US*, n_2_ = 40 ^*a*^	
Right mandibular head				
normal outline	40	22 (55.00%)	31 (77.50%)	0.039
abnormal outline	40	18 (45.00%)	9 (22.50%)	0.039
erosions present	40	7 (17.50%)	3 (7.50%)	0.289
osteophytes present	40	11 (27.50%)	3 (7.50%)	0.027
Left mandibular head				
normal outline	40	27 (67.50%)	23 (57.50%)	0.502
abnormal outline	40	13 (32.50%)	10 (25.00%)	0.628
erosions present	40	6 (15.00%)	1 (2.50%)	0.131
osteophytes present	40	5 (12.50%)	8 (20.00%)	0.505
Bone structure				
normal (right)	40	36 (90.00%)	30 (75.00%)	0.149
normal (left)	40	35 (87.50%)	30 (75.00%)	0.182
Conclusions				
degenerative change (right joint)	40	19 (47.50%)	18 (45.00%)	1.000
degenerative change (left joint)	40	14 (35.00%)	22 (55.00%)	0.043

*^a^ n* (%); *^b^* McNemar’s Chi-squared test for count data.

**Table 3:** Comparison of inter-modality agreement for radiological outcomes in TMJ patients evaluated by CBCT and US techniques.

Characteristic	AC1	SE	CI 95%	*p*
Right mandibular head				
normal outline	0.32	0.16	−0.002, 0.645	0.052
abnormal outline	0.32	0.16	−0.002, 0.645	0.052
erosions present	0.74	0.10	0.549, 0.939	<0.001
osteophytes present	0.65	0.12	0.411, 0.886	<0.001
Left mandibular head				
normal outline	0.06	0.17	−0.289, 0.406	0.734
abnormal outline	0.28	0.17	−0.059, 0.619	0.103
erosions present	0.79	0.08	0.622, 0.961	<0.001
osteophytes present	0.69	0.11	0.470, 0.911	<0.001
Bone structure				
normal (right)	0.58	0.13	0.317, 0.840	<0.001
normal (left)	0.68	0.11	0.448, 0.905	<0.001
Conclusions				
degenerative change (right joint)	0.35	0.15	0.053, 0.654	0.022
degenerative change (left joint)	0.41	0.15	0.112, 0.700	0.008

Note: AC1—Gwet’s AC1 measure of agreement; SE—standard error; CI 95%—confidence interval 95%; *p*—*p*-value of statistical test.

**Table 4:** Diagnostic accuracy metrics for US outcome as a test and CBCT outcome as an observation in the form of a contingency table.

Characteristic	AP	AT	Sens	Spec	PPV	NPV	PLR	NLR	T+O−	T−O+	T+T+	T−T−	CCP
Right mandibular head													
normal outline	0.78 (0.62, 0.89)	0.55 (0.38, 0.71)	0.86 (0.65, 0.97)	0.33 (0.13, 0.59)	0.61 (0.42, 0.78)	0.67 (0.30, 0.93)	1.30 (0.90, 0.87)	0.41 (0.12, 1.41)	0.67 (0.41, 0.87)	0.14 (0.03, 0.35)	0.39 (0.22, 0.58)	0.33 (0.07, 0.70)	0.62 (0.46, 0.77)
abnormal outline	0.22 (0.11, 0.38)	0.45 (0.29, 0.62)	0.33 (0.13, 0.59)	0.86 (0.65, 0.97)	0.67 (0.30, 0.93)	0.61 (0.42, 0.78)	2.44 (0.71, 8.43)	0.77 (0.54, 1.11)	0.14 (0.03, 0.35)	0.67 (0.41, 0.87)	0.33 (0.07, 0.70)	0.39 (0.22, 0.58)	0.62 (0.46, 0.77)
erosions present	0.07 (0.02, 0.20)	0.17 (0.07, 0.33)	0.14 (0.00, 0.58)	0.94 (0.80, 0.99)	0.33 (0.01, 0.91)	0.84 (0.68, 0.94)	2.36 (0.25, 22.54)	0.91 (0.67, 1.25)	0.06 (0.01, 0.20)	0.86 (0.42, 1.00)	0.67 (0.09, 0.99)	0.16 (0.09, 0.99)	0.80 (0.64, 0.91)
osteophytes present	0.07 (0.02, 0.20)	0.28 (0.15, 0.44)	0.18 (0.02, 0.52)	0.97 (0.82, 1.00)	0.67 (0.09, 0.99)	0.76 (0.59, 0.88)	5.27 (0.53, 52.48)	0.85 (0.64, 1.13)	0.03 (0.00, 0.18)	0.82 (0.48, 0.98)	0.33 (0.01, 0.91)	0.24 (0.12, 0.41)	0.75 (0.59, 0.87)
Left mandibular head													
normal outline	0.57 (0.41, 0.73)	0.68 (0.51, 0.81)	0.56 (0.35, 0.75)	0.38 (0.14, 0.68)	0.65 (0.43, 0.84)	0.29 (0.10, 0.56)	0.90 (0.52, 1.56)	1.16 (0.52, 2.59)	0.62 (0.32, 0.86)	0.44 (0.25, 0.65)	0.35 (0.16, 0.57)	0.71 (0.44, 0.90)	0.50 (0.34, 0.66)
abnormal outline	0.25 (0.13, 0.41)	0.32 (0.19, 0.49)	0.23 (0.05, 0.54)	0.74 (0.54, 0.89)	0.30 (0.07, 0.65)	0.67 (0.47, 0.83)	0.89 (0.27, 0.90)	1.04 (0.72, 1,51)	0.26 (0.11, 0.46)	0.77 (0.46, 0.95)	0.70 (0.35, 0.93)	0.33 (0.17, 0.53)	0.57 (0.41, 0.73)
erosions present	0.02 (0.00, 0.13)	0.15 (0.06, 0.30)	0.00 (0.00, 0.46)	0.97 (0.85, 1.00)	0.00 (0.00, 0.97)	0.85 (0.69, 0.94)	0.00 (0.00, n/a)	1.03 (0.97, 1.09)	0.03 (0.00,0.15)	1.00 (0.54,1.00)	1.00 (0.02, 1.00)	0.15 (0.06, 0.31)	0.82 (0.67, 0.93)
osteophytes present	0.20 (0.09, 0.36)	0.12 (0.04, 0.27)	0.40 (0.05, 0.85)	0.83 (0.66, 0.93)	0.25 (0.03, 0.65)	0.91 (0.75, 0.98)	2.33 (0.64, 8.54)	0.72 (0.35, 1.50)	0.17 (0.07, 0.34)	0.60 (0.15, 0.95)	0.75 (0.35, 0.97)	0.09 (0.02, 0.25)	0.78 (0.62, 0.89)
Bone structure													
normal (right)	0.75 (0.59, 0.87)	0.90 (0.76, 0.97)	0.75 (0.58, 0.88)	0.25 (0.01, 0.81)	0.90 (0.73, 0.98)	0.10 (0.00, 0.45)	1.00 (0.55, 1.82)	1.00 (0.17, 5.98)	0.75 (0.19, 0.99)	0.25 (0.12, 0.42)	0.10 (0.02, 0.27)	0.90 (0.55, 1.00)	0.70 (0.53, 0.83)
normal (left)	0.75 (0.59, 0.87)	0.88 (0.73, 0.96)	0.80 (0.63, 0.92)	0.60 (0.15, 0.95)	0.93 (0.78, 0.99)	0.30 (0.07, 0.65)	2.00 (0.67, 5.93)	0.33 (0.13, 0.88)	0.40 (0.05, 0.85)	0.20 (0.08, 0.37)	0.07 (0.01, 0.22)	0.70 (0.35, 0.93)	0.78 (0.62, 0.89)
Conclusions													
degenerative change (right joint)	0.45 (0.29, 0.62)	0.47 (0.32, 0.64)	0.63 (0.38, 0.84)	0.71 (0.48, 0.89)	0.67 (0.41, 0.87)	0.68 (0.45, 0.86)	2.21 (1.04, 4.72)	0.52 (0.27, 0.99)	0.29 (0.11, 0.52)	0.37 (0.16, 0.62)	0.33 (0.13, 0.59)	0.32 (0.14, 0.55)	0.68 (0.51, 0.81)
degenerative change (left joint)	0.55 (0.38, 0.71)	0.35 (0.21, 0.52)	0.86 (0.57, 0.98)	0.62 (0.41, 0.80)	0.55 (0.32, 0.76)	0.89 (0.65, 0.99)	2.23 (0.06, 0.87)	0.23 (0.06, 0.87)	0.38 (0.20, 0.59)	0.14 (0.02, 0.43)	0.45 (0.24, 0.68)	0.11 (0.01, 0.35)	0.70 (0.53,0.83)

Note: AP—apparent prevalence; TP—true prevalence; Sens—sensitivity; Spec—specificity; PPV—positive predictive value; NPV—negative predictive value; PLR—positive likelihood ratio; NLR—negative likelihood ratio; T+O−—CBCT proportion for true observation event; T−O+—false US proportion for true observation event; T+T+—false CBCT proportion for observation event; T−T−—false US no event proportion for US no event; CCP—correctly classified proportion.

## Data Availability

The data on which this study is based will be made available upon request.

## References

[1] Kapos F.P., Exposto F.G., Oyarzo J.F., Durham J. (2020). Temporomandibular disorders: A review of current concepts in aetiology, diagnosis and management. Oral Surg..

[2] Alomar X., Medrano J., Cabratosa J., Clavero J.A., Lorente M., Serra I., Monill J.M., Salvador A. (2007). Anatomy of the temporomandibular joint. Semin. Ultrasound CT MRI.

[3] Bechtold T.E., Kurio N., Nah H.-D., Saunders C., Billings P.C., Koyama E. (2019). The Roles of Indian Hedgehog Signaling in TMJ Formation. Int. J. Mol. Sci..

[4] Sharma S., Gupta D.S., Pal U.S., Jurel S.K. (2011). Etiological factors of temporomandibular joint disorders. Natl. J. Maxillofac. Surg..

[5] Gupta A., Acharya G., Singh H., Poudyal S., Redhu A., Shivhare P. (2022). Assessment of Condylar Shape through Digital Panoramic Radiograph among Nepalese Population: A Proposal for Classification. BioMed Res. Int..

[6] Ahmad M., Schiffman E.L. (2016). Temporomandibular Joint Disorders and Orofacial Pain. Dent. Clin. N. Am..

[7] Wroclawski C., Mediratta J.K., Fillmore W.J. (2023). Recent Advances in Temporomandibular Joint Surgery. Medicina.

[8] Gauer R.L., Semidey M.J. (2015). Diagnosis and treatment of temporomandibular disorders. Am. Fam. Physician.

[9] Zieliński G., Pająk-Zielińska B., Ginszt M. (2024). A Meta-Analysis of the Global Prevalence of Temporomandibular Disorders. J. Clin. Med..

[10] Wojciechowska B., Szarmach A., Michcik A., Wach T., Drogoszewska B. (2024). Association between Clinical Manifestations in Temporomandibular Joint Disorders and Corresponding Radiographic Findings. J. Clin. Med..

[11] Murphy M.K., MacBarb R.F., Wong M.E., Athanasiou K.A. (2013). Temporomandibular disorders: A review of etiology, clinical management, and tissue engineering strategies. Int. J. Oral Maxillofac. Implant..

[12] Mélou C., Pellen-Mussi P., Jeanne S., Novella A., Tricot-Doleux S., Chauvel-Lebret D. (2023). Osteoarthritis of the Temporomandibular Joint: A Narrative Overview. Medicina.

[13] Chung M.K., Wang S., Alshanqiti I., Hu J., Ro J.Y. (2023). The degeneration-pain relationship in the temporomandibular joint: Current understandings and rodent models. Front. Pain Res..

[14] Gandhi V., Sharma G., Dutra E.H., Chen P.J., Yadav S. (2024). Degenerative disorders of temporomandibular joint—Current practices and treatment modalities. Semin. Orthod..

[15] Tanaka E., Detamore M.S., Mercuri L.G. (2008). Degenerative disorders of the temporomandibular joint: Etiology, diagnosis, and treatment. J. Dent. Res..

[16] Feng S.Y., Lei J., Li Y.X., Shi W.G., Wang R.R., Yap A.U., Wang Y.X., Fu K.Y. (2022). Increased joint loading induces subchondral bone loss of the temporomandibular joint via the RANTES-CCRs-Akt2 axis. JCI Insight.

[17] Yap A.U., Lei J., Zhang X.H., Fu K.Y. (2023). TMJ degenerative joint disease: Relationships between CBCT findings, clinical symptoms, and signs. Acta Odontol. Scand..

[18] Kothari S.F., Bead-Hansen L., Hansen L.B., Bang N., Sørensen L.H., Eskildsen H.W., Svensson P. (2016). Pain profiling of patients with temporomandibular joint arthralgia and osteoarthritis diagnosed with different imaging techniques. J. Headache Pain.

[19] Bag A.K., Gaddikeri S., Singhal A., Hardin S., Tran B.D., Medina J.A., Curé J.K. (2014). Imaging of the temporomandibular joint: An update. World J. Radiol..

[20] Larheim T.A., Hol C., Ottersen M.K., Mork-Knutsen B.B., Arvidsson L.Z. (2018). The Role of Imaging in the Diagnosis of Temporomandibular Joint Pathology. Oral Maxillofac. Surg. Clin. N. Am..

[21] Dhabale G.S., Bhowate R.R. (2022). Cone-Beam Computed Tomography for Temporomandibular Joint Imaging. Cureus.

[22] Palconet G., Ludlow J.B., Tyndall D.A., Lim P.F. (2012). Correlating cone beam CT results with temporomandibular joint pain of osteoarthritic origin. Dentomaxillofac. Radiol..

[23] De Nordenflycht D., Tesch R.S. (2022). Advantages of ultrasound guidance for TMJ arthrocentesis and intra-articular injection: A narrative review. Dent. Med. Probl..

[24] Schiffman E., Ohrbach R., Truelove E., Look J., Anderson G., Goulet J.P., List T., Svensson P., Gonzalez Y., Lobbezoo F. (2014). Diagnostic Criteria for Temporomandibular Disorders (DC/TMD) for Clinical and Research Applications: Recommendationsof the International RDC/TMD Consortium Network* and Orofacial Pain Special Interest Group. J. Oral Facial Pain Headache.

[25] Zaman M.U., Alam M.K., Alqhtani N.R., Alqahtani M., Alsaadi M.J., Ronsivalle V., Cicciù M., Minervini G. (2024). Effectiveness of ultrasonography in the diagnosis of temporomandibular joint disorders: A systematic review and meta-analysis. J. Oral Rehabil..

[26] Manfredini D., Guarda-Nardini L. (2009). Ultrasonography of the temporomandibular joint: A literature review. Int. J. Oral Maxillofac. Surg..

[27] Halligan S., Altman D.G., Mallett S. (2015). Disadvantages of using the area under the receiver operating characteristic curve to assess imaging tests: A discussion and proposal for an alternative approach. Eur. Radiol..

[28] Chęciński M., Chęcińska K., Bliźniak F., Lubecka K., Turosz N., Rąpalska I., Michcik A., Chlubek D., Sikora M. (2024). Temporomandibular Joint (TMJ) Replacement Affects Quality of Life: A Systematic Review and Synthesis of Clinical Trials. Appl. Sci..

[29] Lubecka K., Chęcińska K., Bliźniak F., Chęciński M., Turosz N., Michcik A., Chlubek D., Sikora M. (2024). Intra-Articular Local Anesthetics in Temporomandibular Disorders: A Systematic Review and Meta-Analysis. J. Clin. Med..

[30] Klatkiewicz T., Gawriołek K., Pobudek Radzikowska M., Czajka-Jakubowska A. (2018). Ultrasonography in the Diagnosis of Temporomandibular Disorders: A Meta-Analysis. Med. Sci. Monit..

[31] Hussain A.M., Packota G., Major P.W., Flores-Mir C. (2008). Role of different imaging modalities in assessment of temporomandibular joint erosions and osteophytes: A systematic review. Dentomaxillofac. Radiol..

[32] Almashraqi A.A., Sayed B.A., Mokli L.K., Jaafari S.A., Halboub E., Parveen S., Al-Ak’hali M.S., Alhammadi M.S. (2024). Recommendations for standard criteria for the positional and morphological evaluation of temporomandibular joint osseous structures using cone-beam CT: A systematic review. Eur. Radiol..

[33] Al-Saleh M.A., Alsufyani N.A., Saltaji H., Jaremko J.L., Major P.W. (2016). MRI and CBCT image registration of temporomandibular joint: A systematic review. J. Otolaryngol. Head Neck Surg..

